# Evaluation of Laboratories Supporting Invasive Bacterial Vaccine-Preventable Disease (IB-VPD) Surveillance in the World Health Organization African Region, through the Performance of Coordinated External Quality Assessment

**DOI:** 10.3390/tropicalmed8080413

**Published:** 2023-08-14

**Authors:** Inacio Mandomando, Jason M. Mwenda, Tomoka Nakamura, Linda de Gouveia, Anne von Gottberg, Brenda A. Kwambana-Adams, Martin Antonio, Augusto Messa, David Litt, Shila Seaton, Goitom Gebremedhin Weldegebriel, Joseph Nsiari-Muzeyi Biey, Fatima Serhan

**Affiliations:** 1 Centro de Investigação em Saúde de Manhiça (CISM), Maputo P.O. Box 1929, Mozambique; inacio.mandomando@manhica.net (I.M.);; 2 Instituto Nacional de Saúde (INS), Maputo P.O. Box 3943, Mozambique; 3 ISGlobal, Hospital Clínic, Universitat de Barcelona, 08036 Barcelona, Spain; 4 World Health Organization (WHO), Regional Office for Africa, Brazzaville P.O. Box 06, Congo; 5 Department of Infectious Disease Epidemiology, Faculty of Epidemiology and Population Health, London School of Hygiene and Tropical Medicine, London WC1E 7HT, UK; tomoka.nakamura@lshtm.ac.uk; 6 School of Tropical Medicine and Global Health, Nagasaki University, Nagasaki 852-8523, Japan; 7 Department of Immunization, Vaccines and Biologicals, World Health Organization, 1202 Geneva, Switzerland; serhanfa@who.int; 8 Centre for Respiratory Diseases and Meningitis, National Institute for Communicable Diseases, National Health Laboratory Service, Johannesburg 2131, South Africa; lindad@nicd.ac.za (L.d.G.); annev@nicd.ac.za (A.v.G.); 9 Medical Research Council Unit The Gambia at the London School of Hygiene and Tropical Medicine, Banjul P.O. Box 273, The Gambia; brenda.kwambana@lstmed.ac.uk (B.A.K.-A.); martin.antonio@lshtm.ac.uk (M.A.); 10 Department of Clinical Sciences, Liverpool School of Tropical Medicine, Liverpool L7 8XZ, UK; 11 Department of Infection Biology, Faculty of Infectious and Tropical Diseases, London School of Hygiene and Tropical Medicine, London WC1E 7HT, UK; 12 Centre for Epidemic Preparedness and Response, London School of Hygiene and Tropical Medicine, London WC1E 7HT, UK; 13 Respiratory and Vaccine Preventable Bacteria Reference Unit, United Kingdom Health Security Agency (Formerly Public Health England), London NW9 5EQ, UK; david.litt@ukhsa.gov.uk; 14 World Health Organization Collaborating Centre for Haemophilus Influenzae and Streptococcus Pneumoniae, United Kingdom Health Security Agency (Formerly Public Health England), London SW1P 3JR, UK; 15 United Kingdom National External Quality Assessment Service (UK NEQAS) for Microbiology, United Kingdom Health Security Agency (Formerly Public Health England), London NW9 1GH, UK; shila.seaton@ukhsa.gov.uk; 16 World Health Organization (WHO), Inter Country Support Team (IST), Harare P.O. Box 5160, Zimbabwe; weldegebrielg@who.int; 17 World Health Organization (WHO), Inter Country Support Team (IST), Ouagadougou 03 BP 7019, Burkina Faso; bieyj@who.int

**Keywords:** EQA, vaccine-preventable disease, *Streptococcus pneumoniae*, *Neisseria meningitidis*, *Haemophilus influenzae*

## Abstract

(1) Background: Laboratories supporting the invasive bacteria preventable disease (IB-VPD) network are expected to demonstrate the capacity to identify the main etiological agents of pediatric bacterial meningitis (PBM) (*Neisseria meningitidis, Streptococcus pneumoniae* and *Haemophilus influenzae*) on Gram stains and in phenotypic identification. Individual reports of sentinel site (SSL), national (NL) and regional reference (RRL) laboratories participating in the World Health Organization (WHO)-coordinated external quality assessment, distributed by the United Kingdom National External Quality Assessment (EQA) Services (UK NEQAS) for Microbiology between 2014 and 2019 were analyzed. (2) Methods: The panels consisted of (1) unstained bacterial smears for Gram staining, (2) viable isolates for identification and serotyping/serogrouping (ST/SG) and (3) simulated cerebral spinal fluid (CSF) samples for species detection and ST/SG using polymerase chain reaction (PCR). SSLs and NLs tested for Gram staining and species identification (partial panel). RRLs, plus any SSLs and NLs (optionally) also analyzed the simulated CSF samples (full panel). The passing score was ≥75% for NLs and SSLs, and ≥90% for RRLs and NLs/SSLs testing the full panel. (3) Results: Overall, 63% (5/8) of the SSLs and NLs were able to correctly identify the targeted pathogens, in 2019; but there were challenges to identify *Haemophilus influenzae* either on Gram stains (35% of the labs failed 2014), or in culture. Individual performance showed inconsistent capacity, with only 39% (13/33) of the SSLs/NLs passing the EQA exercise throughout all surveys in which they participated. RRLs performed well over the study period, but one of the two failed to reach the minimal passing score in 2016 and 2018; while the SSLs/NLs that optionally tested the full panel scored between 75% and 90% (intermediate pass category). (4) Conclusions: We identified a need for implementing a robust quality management system for timely identification of the gaps and then implementing corrective and preventive actions, in addition to continuous refresher training in the SSLs and NLs supporting the IB-VPD surveillance in the World Health Organization, Regional Office for Africa (WHO AFRO).

## 1. Introduction

Sub-Saharan African countries experience a high burden of childhood mortality due to infectious diseases, with invasive bacterial diseases such as pneumonia, sepsis and meningitis contributing a substantial proportion [1,2]. However, because of limited bacteriology laboratory capacity in many African countries, ascertaining the pathogen-specific attributable burden remains a challenge. Previous studies have documented that *Haemophilus influenzae* type b (Hib), *Neisseria meningitidis* (meningococcus), *Streptococcus pneumoniae* (pneumococcus), non-typhoidal *Salmonella*, *Klebsiella pneumoniae*, *Staphylococcus aureus* and *Escherichia coli* are among the leading bacterial pathogens causing pediatric meningitis and sepsis/bacteremia, often associated with a high case fatality rate [3,4]. Among the laboratories with microbiology capacities, inconsistency remains one of the major issues, partly due to non-compliance with quality assurance standards. It is crucial to generate high quality data of disease burden in the African Region for supporting the introduction and/or monitoring the impact of new vaccines targeting *H. influenzae*, *N. meningitidis* and *S. pneumoniae*.

Laboratory quality assurance management systems include external quality assessments (EQAs), which are unbiased assessments of detecting non-conformances and implementation of corrective actions as part of continuous quality improvement [5]. As part of the efforts to strengthen the capacity of public health laboratories, particularly for testing pathogens that cause epidemic-prone diseases in the World Health Organization (WHO) Regional Office for Africa (AFRO), EQA annual surveys were distributed across the region [6,7,8]. EQA is a critical component for assessing a laboratory’s performance, thus ensuring these laboratories provide consistent high-quality test results. Since 2002, the WHO Regional Office for Africa (AFRO) has been coordinating the pediatric bacterial meningitis (PBM) surveillance network in some African countries [9], and in 2008 it was incorporated as part of the WHO Global Invasive Bacterial Vaccine-Preventable Diseases (IB-VPD) Surveillance Network [10]. This network comprises hospital sentinel site laboratories (SSLs), national laboratories (NLs) and regional reference laboratories (RRLs). National laboratories supporting the PBM surveillance are expected at the very minimum to have capacity for bacterial culture and accurate identification of targeted bacterial pathogens (*H. influenzae*, *N. meningitidis* and *S. pneumoniae*), and additionally, at RRL level, to perform molecular detection and serotyping of these pathogens. In this regard, the WHO AFRO in collaboration with the National Institute for Communicable Diseases (NICD) in Johannesburg, South Africa, launched a Regional Microbiology EQA Program for national public health and other laboratories in the African Region in 2002 [11]. The aim of this program was initially to assess the capacity of public health laboratories to detect endemic and epidemic-prone meningitis-related bacterial pathogens including *H. influenzae, *N. meningitidis** and *S. pneumoniae* and was later extended to other bacterial pathogens [8,11]. The program consisted of shipments of EQA materials, three times annually, to National Public Health Laboratories (NHPLs) (and/or the main hospital or research laboratories functioning as NPHLs), as well as laboratories involved in the PBM surveillance network. The aim was to provide proficiency in testing samples to microbiology laboratories for detection of important pathogens that cause infectious diseases on the African continent, and to monitor participant performance and identify problems. EQA panels consisted of clinically relevant simulated preparations, such as cerebrospinal fluid (CSF) smears for Gram staining and simulated biological fluids (CSF, stool and pus) inoculated with appropriate bacteria, including potential pathogens and associated normal flora. Surveys were sent to participating and referee laboratories simultaneously [11]. Referee laboratories were used to control the quality of the EQA material and their responses were evaluated before determining evaluation criteria within each grading area for participating laboratories. The EQA panels covered bacterial enteric diseases, bacterial meningitis, general bacteriology (blood culture, swabs, etc.), antimicrobial susceptibility testing, plague, malaria microscopy and acid-fast bacilli (*Mycobacterium* spp.) microscopy. In 2011, this EQA program was expanded to include other laboratories in the WHO AFRO participating in the IB-VPD, formerly PBM surveillance network, and it was run until 2013. 

In order to support the Global IB-VPD surveillance network, from 2014, the United Kingdom National External Quality Assessment Services (UK NEQAS) for Microbiology and the national reference laboratories of Public Health England (PHE) were identified as WHO collaborators/partners to implement an annual microbiology EQA to assess the capacity of bacteriology laboratories across all WHO regions, including the Regional Office for Africa. As part of efforts to support the laboratories in Africa in maintaining high standards in microbiology laboratories serving PBM surveillance in the WHO AFRO, the WHO has been coordinating technical hands-on training workshops including on-site assessments to evaluate the progress in implementing the guidelines and identify areas for improvement [12,13]. On the other hand, since 2009, the African Society for Laboratory Medicine (ASLM) has been very active in trying to improve health laboratories’ priorities by strengthening laboratory capacity on the continent, and many countries may have been enrolled in Stepwise Laboratory (Quality) Improvement Process Towards Accreditation (SLIPTA) or Strengthen Laboratory Management Towards Accreditation (SLMTA) endeavors [14,15,16]. Despite all these efforts, the COVID-19 pandemic contributed to the disruption of many services including distribution of EQA panels, but many laboratories have implemented molecular techniques (polymerase chain reaction—PCR) to respond to the COVID-19 pandemic, which may benefit the IB-VPD surveillance network (if the required consumables are available) and subsequent testing of their samples using PCR and/or culture. 

The performance of laboratories supporting the IB-VPD surveillance at a global level was recently published, highlighting the progress and challenges in identifying meningitis-prone pathogens [17]. However, in that report no regional evaluation was conducted to assess the impact that EQAs had on participating African laboratories and whether they were meeting the priority-defined objectives of improving laboratory performance. Therefore, assessing the performance of each individual laboratory supporting IB-VPD surveillance in the WHO AFRO is critical to identify areas for improvement and define appropriate corrective actions. Herein, we aimed to retrospectively review individual reports of each laboratory serving the IB-VPD surveillance network in the WHO AFRO through the EQA program to assess individual performance over a six-year period prior to the COVID-19 pandemic, 2014–2019.

## 2. Materials and Methods

The WHO IB-VPD EQA distribution (provided by the UK NEQAS) was conducted once per calendar year, and each full panel of samples consisted of (i) unstained bacterial smears for Gram-staining, (ii) viable isolates for identification and serotyping/serogrouping (ST/SG) and (iii) simulated CSF samples for PCR-based species detection and ST/SG. Details on sample preparation, quality control, shipping and laboratory testing, reporting to the UK NEQAS and results scoring are detailed elsewhere [17]. Briefly, three or four bacterial films on glass slides were included in each distribution for Gram staining. In early distributions, these contained a light suspension of bacteria in simulated CSF solution (containing blood buffy coat); in later distributions some contained heavier suspensions of bacteria resuspended in water to make the analysis simpler. The suspensions were prepared as heat-fixed films. Seven viable bacterial isolates were prepared as freeze-dried pure cultures by the UK NEQAS in collaboration with the PHE Respiratory and Vaccine Preventable Bacteria Reference Unit (RVPBRU) and the PHE Meningococcal Reference Unit (MRU). All samples of viable bacteria were quality controlled to assess their viability, contamination, stability and homogeneity. Pre- and post-freeze-drying testing of the samples was carried out by the PHE reference laboratories, except for the antimicrobial sensitivity testing. Antimicrobial susceptibility testing (following EUCAST guidelines) was conducted by the PHE Antimicrobial Resistance and Healthcare Associated Infections Bacterial Reference Unit (for *S. pneumoniae* and *H. influenzae*), the PHE MRU (for *N. meningitidis*) and the EUCAST Reference Laboratory in Sweden (for all three species). The MIC values generated by these laboratories were used as reference values for data analysis. For the simulated CSF samples (for PCR detection), seven suspensions of heat-killed bacteria were prepared by the MRU and the RVPBRU. They were then diluted in simulated CSF and freeze-dried by the UK NEQAS. The samples were quality controlled to assess their DNA content and stability. The bacterial cell concentrations in the simulated CSF samples were chosen to be representative of routine detection levels in the UK, although providing sufficiently high levels of DNA that PCR serogrouping/typing should be possible. All samples remained stable and uncontaminated beyond the official closing date of the surveys. 

SSLs and NLs were expected to perform Gram staining on the slides provided and identify the isolates (referred to as testing the partial panel). RRLs, and two SSLs/NLs that had the necessary additional laboratory capacity, were also assessed on their ability to serotype or serogroup the isolates and to perform PCR on the simulated CSF samples (referred to as testing the full panel). Participants were encouraged to perform antimicrobial sensitivity testing (AST) of the viable cultures as an optional exercise but results were excluded in the scoring scheme and are not discussed here. Table 1 summarizes the testing steps and components of each test and the expected results. 

The panels primarily contained isolates of *H. influenzae*, *N. meningitidis* and *S. pneumoniae*, as well as other species that may cause bacterial meningitis (*Listeria monocytogenes*, *Streptococcus agalactiae*, *Escherichia coli* and *Neisseria lactamica*) [17]. Panels were prepared by PHE reference laboratories and the UK NEQAS, and shipped to SSLs, NLs and RRLs in all WHO global regions, including the AFRO. Participants submitted their results to the UK NEQAS via an online portal or by email before the closing date. 

The EQA scheme did not specify the exact methodology that each participant should use to analyze the samples. It was assumed that participants would use laboratory methods described in the WHO’s laboratory manual for diagnosis of meningitis caused by *N. meningitidis*, *S. pneumoniae* and *H. influenzae* [18], although other methods were acceptable. If they attempted ST/SG of bacterial isolates or simulated CSF samples, they were expected to be able to obtain the correct serogroup for *N. meningitidis* or serotype for *H. influenzae*; an incomplete result (e.g., containing more than one possible result) was accepted as partially correct. The same rules applied to serotyping of *S. pneumoniae* if performed phenotypically; however, for PCR typing, it was assumed that the participants were using the conventional PCR typing method described in the WHO laboratory manual [18] or the quantitative PCR equivalent designed by the USA CDC [19,20,21,22]; these were only able to generate a partially correct result for some serotypes chosen for the panels. These partially correct answers were accepted as fully correct for the purposes of the EQA.

The scoring scheme for the EQA is described in detail elsewhere [17]. Briefly, any result matching the intended result for the Gram staining results was given a score of “1”; incorrect results were scored as “0”. A correct result for species identification and ST/SG results (for either the viable cultures or the simulated CSFs) was given a score of “2”. Any species identification result that was only correct to genus level was given a score of “0”; a result incorrect at genus level was scored as “−1”. Any ST/SG result achieving a partial match with the intended result was given a score of “1”. Any incorrect ST/SG result was given a score of “0”. In general, failure to report an individual result was given a score of “0”. Within each distribution, some Gram staining results from individual samples or identification results from individual viable cultures were excluded from the scoring scheme if <80% of respondents did not achieve the expected result, in accordance with UK NEQAS accreditation standards (ISO17043) [17]. Each component of the EQA was scored separately and then combined to give an overall score. AST results of the viable cultures were excluded from the scoring scheme. 

The performance (final score) of each responding laboratory was determined by the proficiency in performing various bacteriological tests. All SSLs and NLs were evaluated according to the partial panel testing (passing score of ≥75%), however a few also decided to test the full panel in addition. RRLs tested the full panel and were expected to obtain an overall target score of ≥90% in order to pass; some SSLs and NLs that had molecular (PCR) technique capacities and the ability to serotype cultures and volunteered to test the full panel were scored against their performance with the full panel. They could obtain a (full) passing score of ≥90% (consistent with the RRLs) or an intermediate pass (between 75% and 89%), in acknowledgment that their laboratory capabilities were not expected to be as high as those of RRLs. 

After each EQA exercise, a report was sent to each participant by the UK NEQAS showing their results in the context of all other participants, plus their overall score. A global summary report was also prepared by the PHE reference laboratories in collaboration with the UK NEQAS describing the individual results of all the participants and their scores in detail, along with a very brief summary of the difficulties encountered by each laboratory. This global summary report was sent to all WHO regional offices.

### Statistical Analysis

All participating laboratories and whether they matched the expected response (yes or no) were summarized using frequencies and percentages, presented in descriptive tables and graphs. All analyses were performed using R version 4.1.1

## 3. Results

### 3.1. General Description

**Figure 1:**
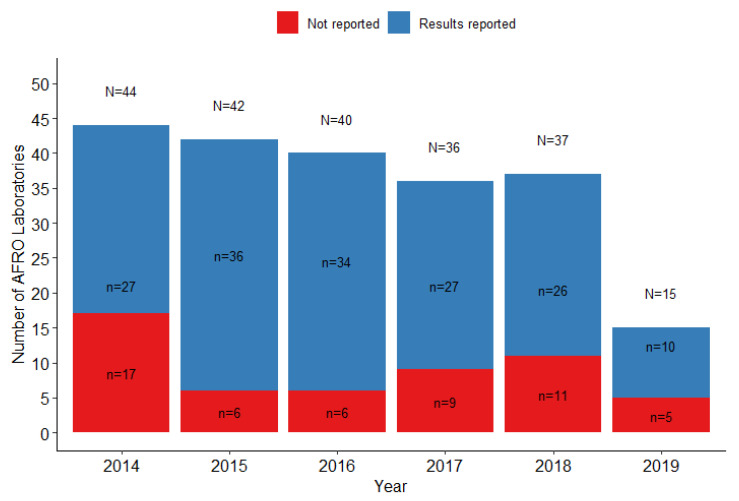
Proportion of WHO AFRO laboratories officially registered with the EQA that reported the results to the UK NEQAS.

During the evaluation period, six panels of EQA were distributed to all WHO AFRO laboratories registered by the WHO with the UK NEQAS. The number of participating laboratories varied each year; and the rate of results returned ranged from 14% (6/42) to 43% (12/40), in 2015 and 2018, respectively. Figure 1 shows the overall total number of laboratories who participated in testing both the partial or full EQA panels between 2014 and 2019, and the percentage of results not returned. The reasons for failure by some laboratories to submit their results were not systematically collected; however, anecdotal reports confirmed that logistical challenges for the shipment of panel samples (including confirmation of correct mailing addresses and communication), problems with customs clearance of panels, and a lack of reagents for sample processing and bacterial identification were contributing factors for many countries.

### 3.2. Performance of Laboratories That Tested the Partial Panel: SSLs and NLs 

**Figure 2:**
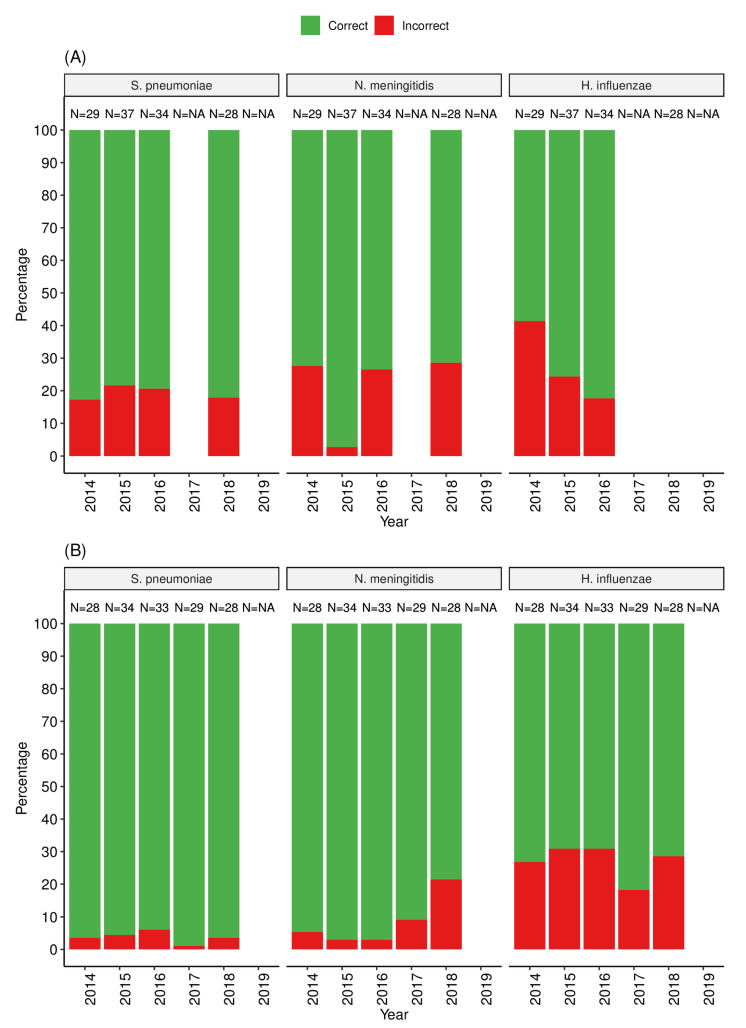
Proportion of SSLs and NLs in the WHO AFRO that correctly identified the targeted pathogens on Gram stains from slide smears (**panel A**) and on viable cultured bacteria (**panel B**).

We assessed the capacity of participating WHO AFRO laboratories to identify the targeted species both by analyzing Gram-stained slides and identifying viable cultured bacteria. The majority of the participants experienced problems in identifying bacterial morphologies compatible with *H. influenzae* on Gram stains, particularly in 2014 and 2017 where <80% of laboratories correctly identified these on Gram stains. Gram staining results for *H. influenzae* were not scored in the EQA scheme for all laboratories in 2014, 2017, 2018 and 2019, because <80% of all (global) participants reported the correct result [17]. In 2014, approximately 40% of the laboratories failed to identify Gram stains, and this proportion decreased to 18% in 2016. Figure 2A shows the proportion of laboratories that correctly identified the three targeted bacteria (*S. pneumoniae*, *N. meningitidis* and *H. influenzae*) on Gram staining. In general, identification of *S. pneumoniae* and *N. meningitidis* was somewhat more successfully than of *H. influenzae*. 

**Figure 3:**
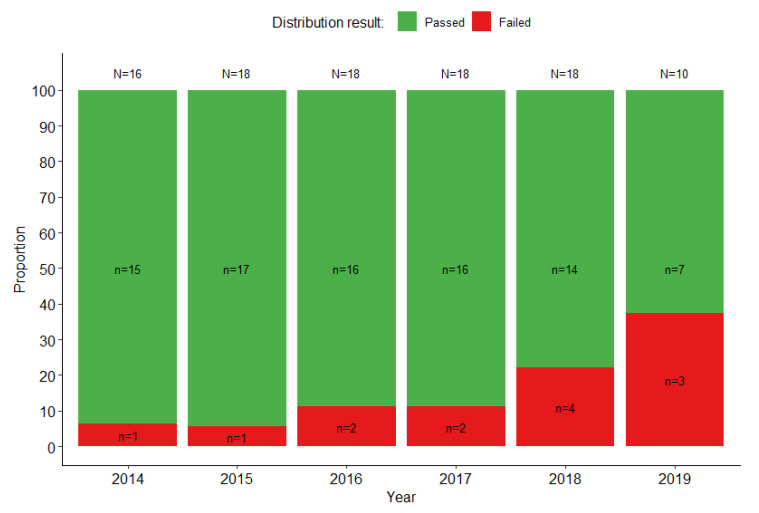
Proportion of SSLs and NLs supporting PBM surveillance in the WHO AFRO that passed the UK NEQAS EQA exercise (≥75% score with the partial panel) each year.

For bacterial culture and identification, similarly to the Gram staining, a higher proportion of SSLs and NLs (up to 35%) failed to identify correctly *H. influenzae* compared to other species (*S. pneumoniae* (<5%) and *N. meningitidis* (<20%)) as shown in Figure 2B. Challenges were also common for SSLs and NLs when identifying other meningitis-causing bacteria in some panels, such as *Listeria monocytogenes*, either using Gram staining or identification of viable cultured bacteria (Supplementary Table S1). An overall performance analysis of the SSLs and NLs that tested partial panels showed that between 63% (5/2) and 94% (17/18) of laboratories passed the EQA exercise (based on Gram staining and culture identification, depending on the year [Figure 3]). The most common errors in correctly identifying *H. influenzae* included incorrect results for Gram staining mostly reported as no bacteria seen or misleading gram positive versus gram negative cocci or coccobacillus, no growth on culture or misidentification and reporting of incorrect species or genus.

**Figure 4:**
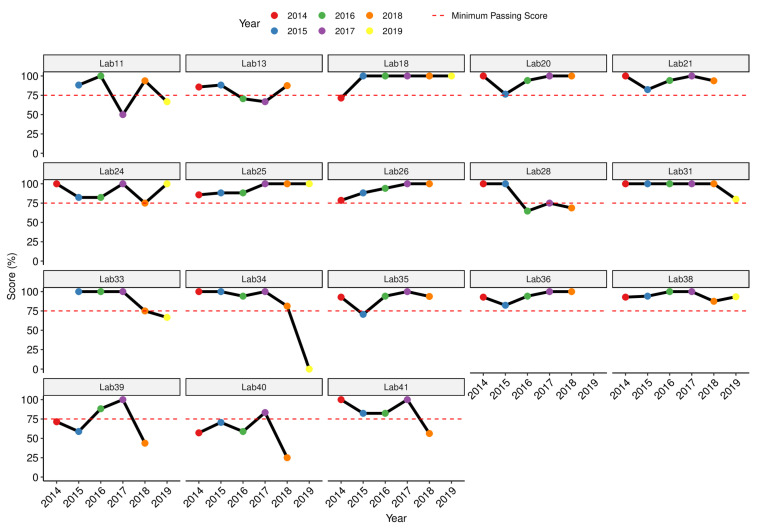
Performance of individual SSLs and NLs that submitted at least three consecutive results for UK NEQAS EQA panels over the years 2014–2019.

We also assessed over the year performance trend for the individual SSLs and NLs for overall scoring, including only laboratories that reported at least three panels (n = 33). The results showed inconsistencies either in reporting the EQA results or passing the exercise (Figure 4). Only 39% (13/33) of the SSLs/NLs passed the EQA exercise throughout all surveys in which they participated (Figure 4). There was a consistent improvement in performance for three laboratories (Lab#25, Lab#26 and Lab#36) reaching a 100% passing score in 2017 and 2018, and in 2019 for Lab#38; while Lab#18 and Lab#31 had excellent performance with passing score of 100% in five of the six EQA panels (Figure 4). 

### 3.3. Performance of Laboratories That Tested the Full Panel: RRLs and Selected SSLs and NLs

**Figure 5:**
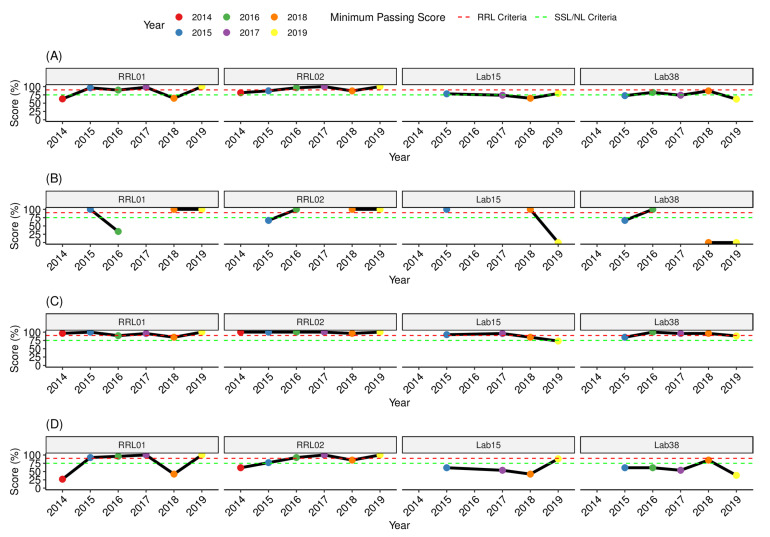
Performance trend of individual RRLs and two SSLs/NLs that tested the UK NEQAS EQA full panel. (**A**) Overall scoring; (**B**) scores for Gram stain only; (**C**) sores for bacterial species identification; (**D**) scores for molecular detection of bacterial species in simulated cerebrospinal fluid only.

The RRLs and two SSLs and NLs (optionally) tested the full panel and their performance was assessed under the criteria of RRL (passing score ≥ 90%). Figure 5 shows the yearly individual performance of the RRLs and SSLs/NLs for overall passing scores (panel A). Single categories (also displayed as percentage of total possible score) are shown for the Gram staining (panel B), viable culture identification and capsule typing (panel C), and simulated cerebrospinal fluid detection and capsule typing (panel D). For overall scoring, one of the two RRLs failed to reach the minimal passing score in 2016 (89%). In contrast, the two SSLs and NLs that tested the full panel failed to reach the 90% passing score when evaluated under the RRL criteria, however, these laboratories consistently ranged between 75% and 90% (which was classified as an intermediate pass), with exception of one year for each (Figure 5A). In various years, the scores for individual Gram stain samples were excluded from the scoring scheme as the overall performance by participants was poor [17] but in general RRLs performed well, as illustrated in Figure 5B.

The two RRLs and one of the SSLs/NLs (Lab#38) showed very good performance passing the EQA exercise with a score of ≥90% in four of the five years testing, Figure 5c, despite some difficulties in identifying bacterial pathogens in simulated CSF (Figure 5d). Nevertheless, the degree of difficulty for accurate etiological diagnosis of simulated CSF was higher for the SSLs and NLs that had a maximum scoring of 89% (once) and in the remaining panels below 70% (Figure 5d). 

Interesting was the fact that the majority of the laboratories testing the partial panel, also attempted the optional ST/SG of the viable cultures, particularly *N. meningitidis* serogrouping, and *H. influenzae* serotyping, but only the two SSLs/NLs that also tested the full panel attempted pneumococcal serotyping (not surprising as pneumococcal serotyping is more complex and costly); not all of them were able to positively confirm the NTHi isolate. In general, some laboratories were very good at identifying the Hib strain, but not good at positively identifying the non-Hib strain (most likely because they only stock the anti-Hib antiserum), as shown in samples Table 1. This EQA also provided opportunity for the laboratories to perform antimicrobial susceptibility testing despite this not being scored, and it is likely that these surveillance platforms can be leveraged to assess other priorities, like antimicrobial susceptibility testing.

## 4. Discussion

Good quality data generated in clinical laboratories are critical to support WHO surveillance activities, which includes monitoring the impact of the introduction of new vaccines. Assessing the performance of laboratories supporting the IB-VPD surveillance is crucial to identify gaps and to suggest relevant actions for improvement. Our data have provided a snapshot on the individual laboratory performance and areas for improvement in the WHO AFRO, where some SSLs, NLs and RRLs performed consistently well for Gram stain and culture identification, but the majority of SSLs/NLs were inconsistent in either result reporting or accurate identification of pathogens targeted by the IB-VPD surveillance, which may have implications for disease burden quantification, clinical management of the patients and monitoring the impact of new vaccines in the region. 

The fact that a high number of laboratories failed to submit their EQA results to the UK NEQAS for at least one survey suggests an urgent need to identify the major factors that drive this for appropriate corrective actions. Reasons for not submitting results to the UK NEQAS may include failure to carry out testing due to lack of or being out of stock of essential reagents, or difficulty with the portal interface to upload the results; however, a full assessment of the reasons for laboratories not reporting the EQA results to the UK NEQAS would have been very valuable. A concerted effort is made by the WHO staff to provide the EQA provider (UK NEQAS) with updated shipping addresses and contact details every year; however, in some cases the delivery is still unsuccessful. Shipping the panels to the WHO country office (WCO) instead of directly to the laboratories may be necessary as a short-term solution to ensure that dispatched panels are ultimately delivered to designated laboratories, while customs and other issues are being addressed as a long-term solution. 

Our findings on the individual performance of SSLs and NLs that tested the partial panel over the years are concerning, because, with the exception of a few laboratories whose performance appeared to be improving over the years, the majority of the laboratories showed inability in their capacity to identify targeted bacteria. Additionally, some laboratories performed well in one survey and then failed on later surveys. Although not systematically collected in this study, factors such as high laboratory staff turnover, procurement challenges, difficulties maintaining adequate stock of laboratory consumables, lack of adequate infrastructures/equipment, and use of inappropriate culture media (e.g., media containing human blood, which can be bactericidal) are some of the major challenges experienced by many laboratories [23,24,25] and may help to explain our findings. Importantly, an assessment of the potential impacts of the inconsistent performance of some laboratories to monitor the impact of IB-VPB surveillance, particularly in quantifying disease burden post vaccine introduction, which were not addressed in this manuscript, are needed. 

The main findings of this analysis demonstrate the need for implementing robust quality management systems for timely identification of the gaps in performance and then implementing the necessary corrective and preventive actions. In addition, continuous refresher training would be valuable, particularly targeting fastidious organisms such as *H. influenzae* as the number of laboratories correctly identifying it on Gram stains was suboptimal compared to the other two pathogens (Figure 2A,B). *H. influenzae* appear as small Gram-negative pleomorphic rods under Gram staining, which are more difficult to identify than *S. pneumoniae* and *N. meningitidis*. Additionally, *H. influenzae* also has more fastidious growth requirements, requiring chocolate agar or the addition of nicotinamide adenine dinucleotide to blood agar for growth [18]. There were some anecdotal reports of Gram staining slides containing very low numbers of bacteria in early EQA distributions that may have contributed to difficulties with Gram staining results. In order to address this, heavier bacterial suspensions were used in later distributions, but this did not solve the problem of generally poorer Gram staining results for *H. influenzae* [17]. On the other hand, limited funds for consistent supply of basic essential laboratory consumables and reagents for accurate detection and identification of pathogens play a major role in the isolation and misidentification of bacterial pathogens, which ultimately generate artificially low bacterial isolation rates [18]. Furthermore, in the African Region, only a limited number of laboratories have regular internal quality control procedures in place, including access to standard reference strains. These are critical for ongoing monitoring of laboratory processes and to correct flaws in the analytical processes of a laboratory before potentially incorrect results of patients are released. They should also result in an improvement in performance in EQA exercises [6,11]. Some of the reasons for underperformance of clinical laboratories in the WHO AFRO are consistent with previous reports (e.g., inadequate supply chain management, lack of skilled personnel and high personnel turnover, poor logistical support and overall lack of quality standards) [6]. Participating laboratories were expected to initiate non-conformances and implement corrective and preventive actions to ensure a structured approach to avoid reoccurring errors. However, it was apparent that participating laboratories did not use their EQA results to identify gaps in their laboratory management systems.

This program has highlighted the need to broaden refresher technical microbiology training for the laboratories supporting the IB-VPD surveillance network in the African Region. Technical skills for laboratory technicians should not focus only on the identification of the three bacterial pathogens causing meningitis (*H. influenzae*, *N. meningitidis* and *S. pneumoniae*) but also include other pathogens of regional and national importance (e.g., *E. coli*, *Streptococcus pyogenes*, *L. monocytogenes*) that have sometimes been misidentified in the EQA panels. Although *L. monocytogenes* is not a common cause of PBM, serious listeriosis outbreaks have been reported in cattle in Nigeria in the 1990s [26], and recently in South Africa, causing more than 1000 cases [27]. On the other hand, it was not surprising that many SSLs and NLs that attempted ST/SG were able to type an Hib culture, as they probably only keep the appropriate antisera but were unable to type any other *H. influenzae* serotypes. For *S. pneumoniae*, most failed to provide a serotype, as many antisera or PCR targets are needed, and this may not be available in many SSLs/NLs because of cost implications [28]. The capacity shown for the SSLs and NLs in serotyping is acceptable for Hib surveillance, but is suboptimal for comprehensive surveillance of *H. influenzae*, calling for further and continuous training.

The two SSLs/NLs that optionally tested the full panels showed acceptable results in terms of serotyping and serogrouping vaccine-related types for the targeted bacterial pathogens (e.g., Hib, MenA and PCV vaccines) [13]. However, there is room for improvement as the results were not consistent over the participating years. Overall, bacteriology laboratories supporting the IB-VPD surveillance network offer additional opportunities for antimicrobial resistance testing in the WHO-AFRO, one of the pressing emerging public health problems [29,30]. Our findings provided a snapshot of areas requiring continuous improvement for the SSLs and NLs for sustainable support of the IB-VPD surveillance network in the WHO AFRO to guide vaccine monitoring, and were critical to support the WHO AFRO in conducting refresher workshop training for countries supporting IB-VPD surveillance organized by the National Institute for Communicable Disease (Johannesburg, South Africa) and the Medical Research Centre, The Gambia Unit, in October and November 2019.

## 5. Study Limitations

Some of the study limitations to be considered in the interpretation of these results include the lack of documentation of factors explaining why some laboratories failed to submit their EQA results to the UK NEQAS or feedback from participants on their non-conformances raised and the resultant corrective actions. 

## 6. Conclusions

Our findings demonstrated a need for implementation of ongoing robust quality management systems for timely identification of gaps and implementation of corrective and preventive actions in all laboratories supporting the IB-VPD surveillance network in the WHO AFRO as a process of continuous improvement of individual laboratory performance. There is a need to create more integrated and sustainable surveillance systems in Africa to support the IB-VPD surveillance including antimicrobial susceptibility testing. 

## Figures and Tables

**Table 1:** Type of samples and testing steps for each distribution panel.

Samples	Test Requested	Intended Result to be Reported (Partial Panel)	Intended Result to be Reported (Full Panel)
Slide smear	Gram staining	Cellular morphologies	
Viable culture	Culture and species identification	Phenotypic or genotypic identification (Serogrouping or serotyping optional and not scored)	Phenotypic or genotypic identification Serogrouping or serotyping *
	Antimicrobial sensitivity testing	MIC results (if available) and S/I/R results, according to each participant’s local guidelines, for a predefined list of appropriate antibiotics (not scored)	
Simulated cerebrospinal fluid (CSF)	PCR testing	(Not included in the partial panel)	Genotypic identification * Genogrouping or genotyping *

* Only with respect to *S. pneumoniae*, *N. meningitidis* or *H. influenzae*.

## Data Availability

Data may be available upon request.

## Supplementary Materials

The following supporting information can be downloaded at: https://www.mdpi.com/article/10.3390/tropicalmed8080413/s1, Table S1: Yearly scoring for Gram stain, bacteria identification (overall score), attempt to serotyping/serogrouping of each laboratory (SSL, NL and RRL) that submitted the UK NEQAS results in participated panel, 2014–2019.
