# Supplementary material for: Evaluation of Laboratories Supporting Invasive Bacterial Vaccine-Preventable Disease (IB-VPD) Surveillance in the World Health Organization African Region, through the Performance of Coordinated External Quality Assessment
Source: Trop Med Infect Dis. 2023 Aug 14;8(8):413. doi: 10.3390/tropicalmed8080413 (PMC10459392; doi:10.3390/tropicalmed8080413)
Supplement: Supplementary file 1 [file tropicalmed-08-00413-s001.zip › tropicalmed-2448118-supplementary.pdf]

Table S1. Yearly scoring for Gram stain, bacteria identification (overall score), attempt to serotyping/serogrouping of each laboratory (SSL, NL and RRL) that submitted the UK NEQAS results in participated panel, 2014–2019

1. 2014

1.1 SSLs/NLs with partial panel

Lab ID	Total score Partial panel	Percentage (Max score = 14)	Comments on problems	Attempted optional serotyping/serogrouping of cultures?			Optional: Gram stain of N. meningitidis. Correct?
				Sp	Nm	Hi	
Lab01	13/14	93%	No problems with Gram stains. Reported the *H. influenzae* culture as "other Haemophilus species"	-	Y	Y	Y
Lab03	14/14	100%	Reported wrong result for Hi Gram stain (not scored).	-	Y	Y	-
Lab09	0/14	0%	This lab did report Gram stain results and got all three samples correct, but didn't test any cultures, so they didn't get any points!	-	-	-	Y
Lab10	14/14	100%	No problems with Gram stains or culture ID.	-	Y	Y	Y
Lab12	14/14	100%	No problems with Gram stains or culture ID.	-	Y	Y	Y
Lab13	12/14	86%	No problems with Gram staining. Reported a *H. influenzae* culture as *N. meningitidis*.	-	Y	Y	Y
Lab15	8/14	57%	No problem with Gram staining. Reported wrong species for a *N. meningitidis* culture and a *S. pneumoniae* culture. Might have been a sample mix-up.	Y	-	Y	Y
Lab16	14/14	100%	No problems with Gram staining or culture ID.	-	Y	Y	Y
Lab18	10/14	71%	No problem with Gram staining. Got no growth for one *H. influenzae* culture. Reported one *S. pneumoniae* culture as the wrong streptococcal species.	-	Y	Y	Y
Lab19	14/14	100%	Reported wrong result for Nm Gram stain (not scored) No problems with culture ID.	-	Y	Y	-
Lab20	14/14	100%	Reported wrong result for Sp and Hi Gram stains (not scored) No problems.	-	Y	Y	Y
Lab21	14/14	100%	Reported wrong result for Hi Gram stain (not scored). No problems with culture ID.	-	Y	Y	Y
Lab22	14/14	100%	Reported wrong result for Hi Gram stain (not scored). No problems with culture ID.	-	Y	Y	Y

Lab24	14/14	100%	Reported wrong result for all 3 Gram stains (not scored). No problems with culture ID.	-	Y	Y	-
Lab25	12/14	86%	No problems with Gram stains (not scored). Could only identify both *H. influenzae* cultures to genus level.	-	-	-	Y
Lab26	11/14	79%	Reported wrong Gram stain result for Hi (not scored). Reported one *H. influenzae* culture as a *N. meningitidis*.	-	-	-	Y
Lab28	14/14	100%	No problems with Gram stains or culture ID.	-	-	-	Y
Lab29	8/14	57% or 80%**	Reported wrong result for all 3 Gram stains (not scored). Reported no growth for one Nm isolate and one Hi isolate. They also did not report any result for the second Hi isolate. **This sample has 2 scores, because in 2014 the denominator was reduced from 14 to 10 for this participant because they reported no growth for 2 samples. From 2015 onwards every lab was tested against the same maximum denominator	-	-	-	-
Lab30	14/14	100%	No problems with Gram stains or culture ID.	-	Y	Y	Y
Lab31	14/14	100%	No problems with Gram stains or culture ID.	-	Y	Y	Y
Lab34	14/14	100%	Reported wrong result for Hi and Nm Gram stains (not scored). No problems with culture ID.	-	Y	Y	-
Lab35	13/14	93%	No problems with Gram stains. Could only identify one of the *H. influenzae* cultures to genus level.	-	Y	Y	Y
Lab36	13/14	93%	No problems with Gram stains. Could only identify one of the *H. influenzae* cultures to genus level.	-	Y	Y	Y
Lab38	13/14	93%	Reported wrong result for Hi and Nm Gram stains (not scored). Could only identify one of the H. influenzae cultures to genus level.	Y	Y	Y	-
Lab39	10/14	71%	No problems with the Gram stains (not scored). Reported one *H. influenzae* culture as *S. pneumoniae* and one *S. pneumoniae* as *H. influenzae* (possibly due to a sample mix-up).	-	-	-	Y
Lab40	8/14	57% or 80%**	Reported wrong result for all 3 Gram stains (not scored). Reported no growth for one *N. meningitidis* culture and one *H. influenzae* culture and did not report a result for the second *H. influenzae* culture. **This sample has 2 scores, because in 2014 the denominator was reduced from 14 to 10 for this participant because they reported no growth for 2 samples. From 2015 onwards every lab was tested against the same maximum denominator	-	Y	-	-
Lab41	14/14	100%	No problems with Gram stains or culture ID.	-	-	-	Y
				**2**	**19**	**19**	**20**

1.2 RRLs testing the Full Panel

Note: in 2014, SSLs/NLs were not invited to test the Full Panel (i.e. they could not test the CSFs and be scored in the same way as the RRLs). The maximum possible score for the Full Panel was 54 points.

Lab ID	ID and typing of cultures score	ID and typing of simulated CSFs score	Total score (%)	Comments
RRL01	**27/28** (96%)	**7/26** (96%)	**34/54** (63%)	Reported wrong result for all 3 Gram stains (not scored). Reported the typing result for the non-typable *H. influenzae* culture as only “not Hib”. This lab also got false negative results for 4 of the 7 simulated CSFs and got a false positive in the negative control sample. *In 2014 the denominator was reduced if a participant didn’t report results for some samples. If the results were scored in the same way as 2015-2018 then the final score would be 63%, but due to this difference this participant is scored as 82%.
RRL02	**28/28** (100%)	**16/26** (77%)	**44/54** (81%)	Reported wrong result for Hi Gram stain (not scored). This lab got false negative results for 2 of the 7 simulated CSFs and couldn’t determine a PCR serotype on one of the *H. influenzae* positive CSFs. * In 2014 the denominator was reduced if a participant didn’t report results for some samples. If the results were scored in the same way as 2015-2018 then the final score would be 81%, but due to this difference this participant is scored as 96%.

2. 2015

2.1 SSLs testing the Partial Panel

Note: The Gram stain result for *L. monocytogenes* was excluded from the scoring in 2015 because many labs got the result wrong. The Gram staining of *L. monocytogenes* was not included in the comments below because only 2 labs got it right.

6 labs did not return any results (no explanation given).

Lab ID	Score	Percentage (Max score = 17)	COMMENTS	Attempted optional serotyping/serogrouping of cultures?		
				**Sp**	**Nm**	**Hi**
Lab01	11/17	**65%**	Incorrect result for Gram stain of Hi. Reported one culture of Hi as negative and the other as Group B Strep.	-	Y	-
Lab02	15/17	**88%**	No problems with Gram-stains. Identified all samples except Group B *Streptococcus* (which they reported as *Streptococcus* species)	-	Y	Y
Lab03	17/17	**100%**	No problems with Gram-staining or species identification	-	Y	Y
Lab04	13/17	**76%**	Problems with Gram-staining of pneumococcus and *H.influenzae and* identification of Group B *Streptococcus* (which they didn't give a result for)	-	Y	Y
Lab06	14/17	**82%**	Problem with Gram-staining of pneumococcus. Failed to identify one of strains of *H.influenzae* (reported it as *Haemophilus* species)	-	Y	Y
Lab07	2/17	**12%**	Problem with Gram-staining of pneumococcus. Did not report <u>any</u> identification of the viable cultures	-	-	-
Lab09	3/17	**18%**	No problems with Gram-staining. Did not report <u>any</u> identification of the viable cultures	-	-	-
Lab11	15/17	**88%**	No problems with Gram-stains. Identified all samples except Group B *Streptococcus* (which they didn't report a result for).	-	Y	Y
Lab12	13/17	**76%**	No problems with Gram-stains. Identified all samples except *H.influenzae* serotype f (which they didn't report a result for) and the Group B *Streptococcus* (which they didn't report a result for).	-	Y	Y
Lab13	15/17	**88%**	No problems with Gram-stains. Identified all samples except *H. influenzae* serotype f (which they reported as *Haemophilus* species).	-	Y	Y
Lab14	11/17	**71%**	Problems with all Gram stains. Failed to identify Group B *Streptococcus* (which they reported as Group A strep.)	-	-	-
Lab15	17/17	**100%**	**Nb. submitted results via Full Panel**<br>No problems with Gram-stains and identified all samples	**Y**	**Y**	**Y**
Lab16	17/17	**100%**	No problems	-	Y	Y
Lab17	15/17	**88%**	No problems with Gram-stains. Failed to identify *H.influenzae* serotype f (reported as *H. parainfluenzae*).	-	Y	Y
Lab18	17/17	**100%**	No problems	-	-	Y
Lab20	13/17	**76%**	No problems with Gram-stains Failed to identify both viable cultures of *H.influenzae* (did not submit a result for either).	-	Y	-

Lab21	14/17	**82%**	No problems with Gram-stains. Identified all samples except Group B Streptococcus (which they reported as *N. meningitidis*)	-	Y	Y
Lab22	16/17	**94%**	Incorrect result for Gram stain of pneumococci. Identified all viable cultures.	-	Y	Y
Lab24	14/17	**82%**	Incorrect result for Gram stain of *H.influenzae.* Failed to identify one strain of pneumococcus (reported it as Negative)	-	Y	Y
Lab25	15/17	**88%**	No problems with Gram staining. Failed to identify *H.influenzae* serotype f (reported it as Negative).	-	Y	Y
Lab26	15/17	**88%**	No problems with Gram-stains. Identified all samples except Group B Streptococcus (reported it as Strep mitis).	-	-	-
Lab27	8/17	**47%**	Problems with Gram-staining *H.influenzae* and pneumococci. Misidentified the species of 1 strain of meningococcus, 1 strain of pneumococcus and 1 strain of *H.influenzae.*	-	-	-
Lab28	17/17	**100%**	No problems	-	-	Y
Lab29	5/17	**29%**	Problems with Gram-staining pneumococci and meningococci. Failed to identify both strains of *H.influenzae*, 1 strain of meningococcus, 1 strain of pneumococcus and 1 Group B streptococcus (reported them all as no growth).	-	-	-
Lab30	16/17	**94%**	Problem with Gram-staining *H.influenzae*, otherwise all results correct	-	Y	Y
Lab31	17/17	**100%**	No problems	-	Y	Y
Lab32	11/17	**65%**	No problems with Gram-staining. Failed to identify both strains of *H.influenzae* (reported one as H. parainfluenzae and the other as Haemophilus species)	-	-	-
Lab33	17/17	**100%**	No problems	-	Y	Y
Lab34	17/17	**100%**	No problems	-	Y	-
Lab35	12/17	**71%**	No problems with Gram staining. Failed to identify both viable cultures of *H.influenzae* (reported one as N. meningitidis and the other as Haemophilus species).	-	Y	-
Lab36	14/17	**82%**	No problems with Gram staining. Failed to identify 1 *H.influenzae* (reported it as N. meningitidis).	-	-	Y
Lab37	11/17	**65%**	Problems with Gram-staining pneumococci and *H. influenzae*. Failed to identify 1 *H.influenzae* (reported it as Negative).	-	-	-
Lab38	16/17	**94%**	**Reported results via Full Panel.** *No report for H .influenzae Gram-stain. Correctly identified all the viable cultures.*	Y	Y	Y
Lab39	10/17	**59%**	No problem with Gram-staining. Failed to identify both cultures of *H.influenzae* (reported one as Negative and the other as N. meningitidis) and the Group B Streptococcus (didn't submit a result).	-	-	-
Lab40	12/17	**71%**	Problem with Gram-staining pneumococci and *H.influenzae*. Failed to identify *H.influenzae* (reported it as Group B streptococcus).	-	Y	Y
Lab41	14/17	**82%**	No problems with Gram-stains. Identified all samples except Group B Streptococcus (reported it as S. pneumoniae).	-	-	-
				**2**	**23**	**22**

2.2 SSL/NL and RRL results with Full Panel:

Based on scoring summary table from 2015 RRL Report.

Note: one RRL failed scoring for getting <90%.

Two SSL/NLs took part in the Full Panel testing. One got an intermediate pass and the other fails.

Lab No.	Gram stains score	ID and typing of cultures score	ID and typing of simulated CSFs score	Total score (%)	Comments
RRL01	**3/3** (100%)	**26/26** (100%)	**24/26** (92%)	**53/55** (96%)	Did not report a PCR serotyping result for the NTHi CSF sample (even though they did detect Hi). Stated that the concentration of DNA was too low.
RRL02	**2/3** (67%)	**26/26** (100%)	**20/26** (77%)	**48/55** (87%)	Reported incorrect result for *H. influenzae* Gram stain. Reported a false negative PCR result for 1 *N. meningitidis* CSF sample. Also did not report a PCR genogrouping result for the other *N. meningitidis* CSF sample.
Lab15	**3/3** (100%)	**24/26** (92%)	**16/26** (62%)	**43/55** (78%)	Unable to serogroup one of the Nm isolates. Reported false negative result for the 2 Nm CSF samples.
Lab38	**2/3** (100%)	**22/26** (85%)	**16/26** (62%)	**40/55** (73%)	Reported incorrect Gram stain result for *H. influenzae*. Reported incomplete serogrouping results for the two Nm cultures and one Sp culture. Did not report PCR serogrouping or serotyping results on Nm and Hi CSF samples.

3. 2016

3.1 SSLs/NLs testing the Partial Panel

One Gram stain sample (E. coli) was excluded from the scoring in 2016 due to poor identification.

Nb. 5 labs were sent a partial panel, but did not return any results (no explanation). 1 lab reported that they didn't didn't receive the panel and so couldn't take part.

Lab ID	Score	Percentage (Max score = 17)	COMMENTS	Attempted optional serotyping/serogrouping of cultures?		
				Sp	Nm	Hi
Lab01	11/17	65%	This lab made an error with the *N. meningitidis* Gram stain (reporting it Gram-positive instead of Gram-negative). They misidentified 1 *S. pneumoniae* as a Group B *Streptococcus* and 1 *H. influenzae* as a *S. pneumoniae*. Unfortunately, they failed, with a score of 65%. Reported wrong result for E. coli Gram stain (not scored).<br>This laboratory commented that they suffered from a lack of reagents, including antibiotic discs, culture media (nutrient broth) and discs for the oxidase test. They requested technical and material support from WHO.	-	Y	Y
Lab02	17/17	100%	This lab made no errors with culture identification and passed with a score of 100%. Reported wrong result for *E. coli* Gram stain (not scored).	-	Y	Y
Lab03	17/17	100%	This lab made no errors with Gram staining or culture identification and passed with a score of 100%.	-	Y	Y
Lab04	15/17	88%	This lab made no errors with Gram staining. They failed to return a result for sample 3659 (NTHi), but all other culture identification was correct. They passed with a score of 88%.<br>This laboratory commented that they suffered from a lack of reagents, including antibiotic discs. Reported wrong result for E. coli Gram stain (not scored).	-	Y	Y
Lab05	15/17	88%	This lab misreported the *S. pneumoniae* Gram stain as a Gram-negative bacillus and the *N. meningitidis* Gram stain as a Gram-positive coccus. However, they made no errors in the culture identification. They passed with a score of 88%. Reported wrong result for E. coli Gram stain (not scored).	-	Y	Y
Lab06	16/17	94%	This lab did not submit a result for the *S. pneumoniae* Gram stain sample. However, they made no errors in the culture identification. They passed with a score of 94%. Reported wrong result for E. coli Gram stain (not scored).	-	-	Y
Lab07	3/17	18%	This lab reported the three scored Gram stain samples correctly but did not submit any culture identification results. Hence, they failed, with a score of 18%. Reported wrong result for E. coli Gram stain (not scored).	-	-	Y
Lab09	2/17	12%	This lab misreported the *N. meningitidis* Gram stain as a Gram-positive coccus. They also failed to submit any culture identification results. Hence, they failed, with a score of 12%.<br>This laboratory commented that they were unable to analyse the cultures due to a lack of equipment and reagents. Reported wrong result for E. coli Gram stain (not scored).	-	-	Y

Lab11	**17/17**	**100%**	This lab made no errors with Gram staining or culture identification and passed with a score of 100%.	-	Y	-
Lab13	**12/17**	**71%**	This lab misreported the *H. influenzae* Gram stain sample as a Gram-positive bacillus, the *S. pneumoniae* Gram stain sample as a Gram negative coccus/diplococcus, and the *N. meningitidis* Gram stain sample as a Gram-positive coccus/diplococcus. Their culture identification was generally correct, although they could only identify the NTHi culture to genus level. Unfortunately, they failed, with a score of 71%.	-	Y	Y
Lab14	**16/17**	**94%**	This lab made no errors with Gram staining or culture identification, although they failed to report a result for the *S. pneumoniae* Gram stain sample. They passed, with a score of 94%. Reported wrong result for *E. coli* Gram stain (not scored).	-	-	Y
Lab18	**17/17**	**100%**	This lab made no errors with Gram staining or culture identification and passed with a score of 100%. Reported wrong result for *E. coli* Gram stain (not scored).	-	-	Y
Lab20	**16/17**	**94%**	This lab only made 1 error – misreporting the H. influenzae Gram stain sample as a Gram-positive coccus/diplococcus. They passed, with a score of 94%. Reported wrong result for *E. coli* Gram stain (not scored).	-	Y	Y
Lab21	**16/17**	**94%**	This lab only made 1 error – misreporting the *S. pneumoniae* Gram stain sample as a Gram-negative bacillus/coccobacillus. They passed, with a score of 94%. Reported wrong result for *E. coli* Gram stain (not scored).	-	-	Y
Lab22	**0/17**	**0%**	This lab reported "not evaluated" for Gram staining and culture identification, and, hence, scored 0 in the EQA. They did submit some AST results. Reported wrong result for E. coli Gram stain (not scored).	-	-	Y
Lab23	**13/17**	**76%**	This lab made no errors with the Gram staining or culture identification but failed to report identification results for sample 3658 (*N. meningitidis*) and sample 3660 (*S. pneumoniae*). They passed, with a score of 76%. Reported wrong result for *E. coli* Gram stain (not scored).	-	-	Y
Lab24	**14/17**	**82%**	This lab made 1 error with the scored Gram staining samples – misreporting the *H. influenzae* sample as Gram-positive bacillus. They made no errors with culture identification but failed to report a result for sample 3655 (*S. pneumoniae*). This lab commented that they did not report a result for sample 3655 because it became contaminated. They passed, with a score of 82%.	-	-	Y
Lab25	**15/17**	**88%**	This lab made no errors with the scored Gram staining samples. They only had a problem with the identification of 1 culture – only reporting one of the *H. influenzae* samples to genus level. They passed, with a score of 88%. Reported wrong result for *E. coli* Gram stain (not scored).<br>This lab commented that they could not carry out antibiotic sensitivity testing as the cultures lost viability after the identification procedures.	-	-	Y
Lab26	**16/17**	**94%**	This lab only made 1 error – misreporting the *H. influenzae* Gram staining sample as a Gram-positive coccus/diplococcus. They passed, with a score of 94%.	-	-	Y
Lab27	**11/17**	**65%**	This lab did not report any Gram staining results. They only made one error in the culture identification part – misreporting sample 3658 (*N. meningitidis*) as a *H. influenzae*. Unfortunately, they failed, with a score of 65%. Reported wrong result for E. coli Gram stain (not scored).	-	Y	Y
Lab28	**11/17**	**65%**	This lab made 1 error with the Gram staining samples – reporting the *S. pneumoniae* sample as a Gram-negative bacillus/coccobacillus. They also made 1 error with the culture identification, reporting sample 3658 (*N. meningitidis*) as a *S. pneumoniae*, and reported a negative result for sample 3659 (*H. influenzae*). They failed, with a score of 65%.	-	Y	Y

Lab29	**17/17**	**100%**	This lab made no errors with Gram staining or culture identification and passed with a score of 100%. Reported wrong result for *E. coli* Gram stain (not scored).<br>This laboratory commented that the cultures of *H. influenzae* and *N. meningitidis* did not grow on their antibiotic sensitivity testing media.	-	Y	Y
Lab30	**14/17**	**82%**	This lab made no errors with the Gram staining samples. They only made one error with the culture identification – misreporting one of the *N. meningitidis* samples as a *H. influenzae*. They passed, with a score of 82%.	-	Y	Y
Lab31	**17/17**	**100%**	This lab made no errors with Gram staining or culture identification and passed with a score of 100%.	-	Y	Y
Lab32	**15/17**	**88%**	This lab made no errors with Gram staining or culture identification but did report a negative result for sample 3659 (*H. influenzae*). They passed with a score of 88%. Reported wrong result for E. coli Gram stain (not scored).	-	Y	Y
Lab33	**17/17**	**100%**	This lab made no errors with Gram staining or culture identification and passed with a score of 100%.<br>This laboratory commented that they couldn't process the samples immediately because they were lacking nutrient broth.	-	Y	Y
Lab34	**16/17**	**94%**	This lab only made 1 error – misreporting the *N. meningitidis* Gram staining sample as a Gram-positive coccus. They had no problems with culture identification. They passed, with a score of 94%. Reported wrong result for E. coli Gram stain (not scored).	-	Y	Y
Lab35	**16/17**	**94%**	This lab only made 1 error – misreporting the *N. meningitidis* Gram staining sample as a Gram-positive coccus/diplococcus. They had no problems with culture identification. They passed, with a score of 94%.	-	Y	-
Lab36	**16/17**	**94%**	This lab only made 1 error – misreporting the *N. meningitidis* Gram staining sample as a Gram-positive coccus/diplococcus. They had no problems with culture identification. They passed, with a score of 94%.	-	-	Y
Lab37	**2/17**	**12%**	This lab made no errors with the scored Gram staining samples. However, they reported "negative" for both *N. meningitidis* cultures, one of the *H. influenzae* cultures and one of the *S. pneumoniae* cultures. If these were the result of no growth from the sample, it suggests there may be a problem with the agar plate growth medium in this lab. Unfortunately, they also misreported the other two *S. pneumoniae* cultures as Group A *Streptococcus* and Group A *Streptococcus*, and the remaining *H. influenzae* culture as a *N. meningitidis*. These errors may be a problem of culturing due to poor growth media, but they deserve investigation. This lab failed, with a score of 12%.<br>This lab commented that their ability to culture the strains was compromised by a shortage of reagents. Reported wrong result for *E. coli* Gram stain (not scored).	-	-	-
Lab38	17/17	100%	This lab participated in the Full Panel RRL EQA distribution. They had no problems with Gram staining or species identification and would have scored 17/17 (100%) if judged according to this EQA.	Y	-	-
Lab39	**15/17**	**88%**	This lab made no errors with the Gram staining of culture identification samples but failed to report a result for one of the *H. influenzae* samples (3659). (This may have been a failure to grow it. A few other labs reported "negative" for this sample.) They passed, with a score of 88%. Reported wrong result for *E. coli* Gram stain (not scored).	-	-	-

Lab40	**10/17**	**59%**	This lab misreported the *H. influenzae* Gram stain sample as Gram-positive bacillus/coccobacillus, the *S. pneumoniae* Gram stain samples as Gram-negative coccus/diplococcus, and the *N. meningitidis* Gram stain sample as Gram-negative bacillus/coccobacillus They also had a problem with the *H. influenzae* culture identification samples, misreporting one of them as a *H. parainfluenzae*, and the other as "no growth". They failed, with a score of 59%. Reported wrong result for *E. coli* Gram stain (not scored).	-	-	-
Lab41	**14/17**	**82%**	This lab had no problems with the Gram staining samples. They only made 1 error with the culture identification – misreporting one of the *H. influenzae* samples as a *N. meningitidis*. They passed, with a score of 82%. Reported wrong result for E. coli Gram stain (not scored). This laboratory commented that they had no materials for serotyping and no control organisms.	-	-	-
**TOTAL**				**1**	**17**	**27***

*Note: for Hi typing, all the labs marked as "Y" could identify the Hib serotype. However, not all of them were able to positively confirm the NTHi isolate (i.e. some of them did not possess antisera to other serotypes and so could only say that it was "not Hib"; other labs just didn't return a result for the NTHi).

3.2 SSL/NL and RRL results with Full Panel:

Only 1 NL returned results for the Full Panel in 2016. They achieved an intermediate pass.

Nb. WH110 was sent a full panel, but did not return any results. WH115 was sent a full panel and did report that they didn't receive the panel and so couldn't take part.

Lab No.	WHO Region	Lab type	Gram stains	ID and typing of cultures	ID and typing of simulated CSFs	Overall Score (%)	Comments
RRL01	**AFRO**	**RRL**	**1/3 (33%)**	**25/28 (89%)**	**25/26 (96%)**	**51/57 (89%)**	Reported incorrect Gram stain results for Hi and Nm. Reported wrong result for *E. coli* Gram stain (not scored). Reported an incorrect serotype for one of the Sp cultures and reported a partially correct serotype for the other Sp culture. Reported a partially correct serotype for one of the Sp-positive CSF samples.
RRL02	**AFRO**	**RRL**	**3/3 (100%)**	**28/28 (100%)**	**24/26 (92%)**	**55/57 (96%)**	Reported wrong result for *E. coli* Gram stain (not scored). Reported that they couldn't type the NTHi-positive CSF sample due to low DNA concentration. Reported incorrectly the serotype for one of the Sp-positive CSF samples.

Lab38	**AFRO**	**NL**	**3/3 (100%)**	**28/28 (100%)**	**16/26 (62%)**	**47/57 (82%)**	Reported wrong result for *E. coli* Gram stain (not scored). Did not perform PCR capsule typing for Nm or Hi on the CSF samples. Only typed one of the two Sp-positive CSF samples.

4. 2017

Nb. All 3 Gram stains (Hi, Sp and *L. monocytogenes*) plus the *N. lactamica* culture were excluded from the scoring this year.

4.1 SSLs/NLs testing the Partial Panel

Nb. 9 labs were sent a partial panel, but did not report any results (no explanation).

No comments about the *N. lactamica* culture results (not scored) were included in the table below as 3 SSL/NLs got it right.

Lab ID	Score (Max = 12)	Percent	COMMENTS	Attempted optional serotyping/serogrouping of cultures?		
				Sp	Nm	Hi
Lab04	10/12	83%	Reported wrong results for Sp and Lm Gram stain (not scored). Did not return a result for one of the *H. influenzae* isolates, but all other identification results correct.	-	Y	Y
Lab06	10/12	83%	Reported wrong result for Lm Gram stain (not scored). Could only identify one of the *H. influenzae* isolates to genus level, but all other identification results correct.	-	Y	Y
Lab11	6/12	50%	Reported wrong results for Sp and Lm Gram stain (not scored). Misidentified the first two cultures. Did they accidentally swap them?	-	Y	Y
Lab13	8/12	67%	Reported wrong results for Hi Gram stain (not scored). Could not get the two *H. influenzae* isolates to grow. Is there a problem with their growth media?	-	Y	-
Lab15	12/12	100%	This lab tested the Full Panel. They had no trouble with the culture identification and would have scored 100% with the Partial Panel. Reported wrong results for Lm Gram stain (not scored).	Y	Y	Y
Lab16	12/12	100%	Reported wrong results for Lm Gram stain (not scored). No identification problems.	-	Y	Y
Lab17	12/12	100%	Reported wrong results for Hi Gram stain (not scored). No identification problems.	-	-	Y
Lab18	12/12	100%	Reported wrong results for Hi and Lm Gram stain (not scored). No identification problems.	-	Y	Y
Lab20	12/12	100%	Reported wrong results for Sp and Lm Gram stain (not scored). No identification problems.	-	Y	Y
Lab21	12/12	100%	Reported wrong results for Sp and Lm Gram stain (not scored). No identification problems.	-	Y	Y
Lab24	12/12	100%	Reported wrong results for Lm Gram stain (not scored). No identification problems.	-	Y	Y
Lab25	12/12	100%	No problems with Gram stains (not scored). No identification problems.	-	Y	Y
Lab26	12/12	100%	Reported wrong results for Lm Gram stain (not scored). No identification problems.	-	-	-
Lab27	7/12	58%	No problems with Gram stains (not scored). Could not get one of the *H. influenzae* cultures to grow (problem with their media?) and misidentified one of the *N. meningitidis* isolates as a *H. parainfluenzae*.	-	Y	Y
Lab28	9/12	75%	Reported wrong results for Sp and Lm Gram stain (not scored). Misidentified one of the *H. influenzae* cultures as a *S. pneumoniae*.	-	Y	-
Lab30	8/12	67%	Reported wrong results for Sp and Lm Gram stain (not scored). Misidentified one of the *H. influenzae* cultures as *H. haemolyticus* and didn't report a result for one of the *N. meningitidis* cultures.	-	Y	Y

Lab31	12/12	100%	Reported wrong results for Lm Gram stain (not scored). No identification problems.	-	Y	Y
Lab32	12/12	100%	No problems with Gram stains (not scored). No identification problems.	-	-	-
Lab33	12/12	100%	Reported wrong results for Lm Gram stain (not scored). No identification problems.	-	Y	Y
Lab34	12/12	100%	Reported wrong results for Hi and Lm Gram stain (not scored). No identification problems.	-	-	-
Lab35	12/12	100%	Reported wrong results for Lm Gram stain (not scored). No identification problems.	-	Y	Y
Lab36	12/12	100%	Reported wrong results for Lm Gram stain (not scored). No identification problems.	-	Y	Y
Lab37	12/12	100%	Reported wrong results for Hi, Sp and Lm Gram stain (not scored). No identification problems.	-	-	-
Lab38	12/12	100%	This lab tested the Full Panel.<br>Nb. they would have passed the SSL EQA with a score of 100%. Reported wrong results for Sp and Lm Gram stain (not scored).	Y	Y	Y
Lab39	12/12	100%	Reported wrong results for Hi and Lm Gram stain (not scored). No identification problems.	-	-	-
Lab40	10/12	83%	Reported wrong results for Hi, Sp and Lm Gram stain (not scored). Couldn't get one of the *H. influenzae* cultures to grow (problem with growth media?).	-	Y	Y
Lab41	12/12	100%	Reported wrong results for Hi, Sp and Lm Gram stain (not scored). No identification problems.	-	-	-
TOTAL				2	23	19*

*As in previous years, labs are generally very good at identifying the Hib strain, but not good at positively identifying the non-Hib strain (most likely because they only stock the anti-Hib antiserum). Some correctly identified the Hie strain, others just reported it as "not type b" and others just didn't give a result.

4.2 SSL/NL and RRL results with Full Panel:

Lab ID	WHO Region	Gram stains	ID and typing of cultures	ID and typing of simulated CSFs	Overall Score[1] %	Comments
RRL01	AFRO	Not scored	23/24<br>(96%)	26/26<br>(100%)	49/50<br>(98%)	Reported wrong result for *L. monocytogenes* Gram stain (not scored). Only partially serogrouped the Nm W isolate. Reported wrong result for *N. lactamica* culture (not scored).
RRL02	AFRO	Not scored	24/24<br>(100%)	26/26<br>(100%)	50/50<br>(100%)	Reported wrong results for Hi and *L. monocytogenes* Gram stains (not scored). No problems with cultures or simulated CSF samples.
Lab15	AFRO	Not scored	23/24<br>(96%)	14/26<br>(54%)	37/50<br>(74%)	Reported wrong results for Lm Gram stain (not scored). Only partially serogrouped the Nm W isolate. Got false negative results for the Nm-positive CSF samples and they did not report PCR typing results for the Sp-positive CSF samples.

Lab38	**AFRO**	**Not scored**	**23/24**<br>**(96%)**	**14/26**<br>**(54%)**	**37/50**<br>**(74%)**	Reported wrong results for Lm Gram stain (not scored). Only partially serogrouped the Nm W isolate. Did not report any typing results for the non-culture samples. (They misidentified the *N. lactamica* culture – not scored)

Nb. The two SSL/NL labs only just failed to achieve an intermediate pass!

Note: this year there were extra 7 AFRO labs that have not been included in the tables.

5. **2018**

Two Gram stains were excluded from the scoring this year (*L. monocytogenes* and *H. influenzae*).

5.1 SSLs/NLs testing the Partial Panel

11 labs that were sent a Partial Panel, but did not report any results (no reason given).

Lab ID	Score	Percentage	COMMENTS on RESULTS Gram stains and Identification	Attempted optional serotyping/serogrouping of cultures?		
				Sp	Nm	Hi
Lab04	3/16	19%	This lab made several errors with Gram staining. They failed to identify #4902 (*S. pneumoniae*) and #4903 (*Listeria monocytogenes)* on Gram-stained films (calling both Gram-negative). This may be due to over-decolourisation of the films. They also failed to identify # 4904 (*H. influenzae*) on the Gram stain- stating it was Gram-positive cocci/diplococci. The lab misidentified #4906 (NTHi) as *N. meningitidis*. Also misidentified both #4909 (*E.coli*) and #4910 (*N. meningitidis* serogroup C) as Group B *Streptococcus*. They failed to identify # 4911 (Hib), stating it was *Enterobacteria*.. All other culture identification was correct. In the optional typing results, the lab mistyped #4906 (NTHi) as [*N. meningitidis*] Y/W135, #4910 (*N. meningitidis* serogroup C) as Group B Streptococcus. The lab correctly serogrouped #4908 as serogroup A meningococcus.	-	Y	-
Lab11	15/16	94%	This lab made two errors with Gram staining (stating #4904 *H.influenzae* was Gram-negative cocci and # 4905 *N. meningitidis* was Gram-positive cocci). All the culture identifications were correct. Samples #4908 and#4910 (*N. meningitidis* serogroup A and C) were correctly serogrouped.	-	Y	-
Lab12	4/16	25%	This lab failed to identify #4903 (*L. monocytogenes)* on Gram-stain (stating it was Gram-negative bacilli over-decolourisation). They misidentified #4906 (NTHi) as *Neisseria sp.* and misidentified #4908 (*N. meningitidis* serogroup A) as *S. pneumoniae* and misidentified #4910 (*N. meningitidis* serogroup C) as *H. influenzae* and misidentified #4911 (Hib) as *Neisseria.* They reported #4910 (*N. meningitidis* serogroup C) as Hib. It is possible some of the errors were as a result of mixing up the samples, but we cannot be sure that this is the case.	-	-	-
Lab13	14/16	88%	This lab reported the correct result for all of the Gram stains. Their culture identification was generally correct, although they could only identify #4906 (NTHi) as *Haemophilus* unnamed sp. The lab correctly serotyped the *H. influenzae* cultures and correctly serogrouped the meningococcal cultures. They did not report any serotyping Y results for the pneumococcal cultures.	-	Y	Y
Lab15	16/16	100%	This lab tested the Full Panel. However, they would have passed the Partial Panel scoring with 100%. They only reported the *L. monocytogenes* Gram stain result wrong (not scored).	Y	-	Y
Lab16	14/16	88%	This lab reported the correct result for all of the Gram-stains. Culture identification was generally correct although they misidentified #4910 (*N. meningitidis* serogroup C) as *Moraxella ovis*. The lab correctly serotyped #4911 as Hib and #4908 as *N. meningitidis* serogroup A. They did not report any serotyping results for the pneumococcal cultures.	-	Y	Y
Lab18	16/16	100%	This lab made two errors on Gram-staining. They reported #4903 (*L. monocytogenes*) as Gram-negative bacilli/coccobacilli and #4904 (*H. influenzae*) as Gram-negative cocci/diplococci). All of the culture identifications were correct. The lab correctly serotyped #4911 as Hib and #4908 as *N. meningitidis* serogroup A. They did not report any serotyping results for the pneumococcal cultures.	-	Y	Y

Lab20	16/16	100%	This lab incorrectly stated #4903 (L. *monocytogenes*) was Gram-negative bacilli/coccobacilli and #4904 (*H. influenzae*) was Gram-negative cocci/diplococci. All culture identifications were correct. The lab correctly serogropuped #4910 as serogroup C meningococcus but stated #4908 (meningococcus serogroup A) was serogroup X. The lab correctly serotyped #4911 as Hib. They did not report any serotyping results for the pneumococcal cultures.	-	Y	Y
Lab21	15/16	94%	This lab reported #4902 (S.*pneumoniae*) as Gram-negative cocci/ /diplococci. All the culture identifications were correct They reported #4908 (meningococcus serogroup A) was A/Y and #4910 (meningococcus serogroup C) was serogroup W. They correctly serotyped #4911 as Hib. They did not report any serotyping results for the pneumococcal cultures.	-	Y	Y
Lab22	9/16	56%	This lab reported Gram-positive cocci/diplococci for Gram stain #4905 *N. meningitidis.* They misidentified both #4910 (*N. meningitidis* serogroup C) and #4908 (*N. meningitidis* serogroup A) as *H. influenzae.*	-	-	-
Lab24	12/16	75%	This lab made 3 errors in Gram staining. #4903 (*L. monocytogenes*) was wrongly stated to be Gram) negative cocci/diplococci; #4904 (*H. influenzae*) was described as Gram positive bacilli/coccobacilli and #4905 (*N. meningitidis*) was described as Gram negative bacilli/coccobacilli. Sample #4906 (*H. influenzae*) was wrongly identified as *E.coli*; otherwise, the identification of samples was correct. Samples #4908 and #4910 (*N. meningitidis* serogroup A and C) were correctly serogrouped and #4911 was correctly serotyped as Hib.	-	Y	Y
Lab25	16/16	100%	This lab made one error in Gram staining, stating that #4903 *L. monocytogenes* was Gram-negative bacilli/coccobacilli. All of the culture identifications were correct. The lab correctly serogrouped #4908 as meningococcus serogroup A but incorrectly stated #4910 was serogroup Y/W135.They correctly serotyped the Hib strain.	-	Y	Y
Lab26	16/16	100%	This lab made two errors in Gram staining, stating that #4903 (*L. monocytogenes*) was Gram negative bacilli and #4904 (*H. influenzae*) was Gram negative cocci/diplococci. All the identifications of cultures were correct.	-	-	-
Lab27	13/16	81%	This lab made one error in Gram staining, stating that #4903 (*L. monocytogenes*) was Gram-negative bacilli. This lab identified #4906 (NTHi) as *Klebsiella oxytoca*, Samples #4908 and #4910 (*N. meningitidis* serogroup A and C) were correctly serogrouped and #4911 was correctly serotyped as Hib.	-	Y	Y
Lab28	11/16	69%	This lab made two errors in Gram-staining stating that #4903 (*L. monocytogenes*) was Gram-negative bacilli/coccobacilli; and #4904 (*H. influenzae*) was Gram negative cocci/diplococci. They identified culture #4906 (NTHi) as Gram positive bacilli) and did not enter a result for #4911 (Hib)	-	-	-
Lab31	16/16	100%	This lab failed to identify #4903 (*L. monocytogenes*) on Gram-stain stating it was Gram-negative bacilli). All of the cultures were correctly identified. The lab correctly identified #4910 serogroup C meningococcus and #4911 Hib. They did not enter a result for the serogroup of #4908 (serogroup A meningococcus).	-	Y	Y
Lab32	1/16	6%	This lab failed to identify #4903 (*L. monocytogenes*) on Gram-stain, stating it was Gram-negative bacilli), #4904 *H. influenzae)* stating it was Gram-negative cocci/diplococci. They did not examine #4905.They identified #4906 (NTHi) as unnamed *Haemophilus*, #4908 (*N. meningitidis* serogroup A) as *Enterococcus sp.*, #4909 (*E. coli*) as *Haemophilus sp*., #910 (*N. meningitidis*) as unnamed *Neisseria sp.* #4911 (Hib) as unnamed *Haemophilus sp*. and #4912 (*S. pneumoniae*) as unnamed *Streptococcus sp*.<br>Some of these errors were a failure to identify the samples to more than genus level.	-	-	-
Lab33	12/16	75%	This lab failed to identify #4903 (*L.monocytogenes*) on Gram-stain, stating it was Gram negative bacilli) and #4904 H.*influenzae*), stating it was Gram negative cocci/diplococci. They identified # 4906 (NTHi) as unnamed H*aemophilus* and misidentified #4907 (S. *pneumoniae*) as Group B streptococcus. Samples #4908 and# 4910 (*N. meningitidis* serogroup A and C) were correctly serogrouped and #4911 was correctly serotyped as Hib.	-	Y	Y
Lab34	13/16	81%	All Gram-stain results were correct.<br>Sample #4909 (*E. coli*) was misidentified as *Enterobacter agglomerans.*	-	-	-
Lab35	15/16	94%	#4902 (*S. pneumoniae*) was incorrectly stated to be Gram-negative cocci/diplococci; #4903 (*L. monocytogenes*) was stated to be Gram-negative bacilli/coccobacilli. These errors suggest the over-decolourisation of Gram stains. All culture identifications were correct. The lab correctly stated #4911 was Hib.	-	-	Y
Lab36	16/16	100%	The lab incorrectly stated #4903 (*L. monocytogenes*) was Gram-negative bacilli/coccobacilli.; #4904 (*H. influenzae)* was Gram-negative cocci/diplococci. All the cultures were correctly identified.	-	-	Y

Lab38	14/16	88%	This lab tested the Full Panel. However, they would have passed the Partial Panel scoring with 88%. They reported the wrong Gram stain result for the Sp sample and the Hi sample (nb: Hi not scored) and did not report a Gram stain result for the Nm sample. No problem with culture identification.	Y	Y	Y
Lab39	7/16	44%	The lab incorrectly stated #4903 (*L. monocytogenes*) was Gram-negative bacilli/coccobacilli.; #4904 (*H. influenzae)* was Gram negative cocci/diplococci. The lab incorrectly identified #4906 (NTHi) as *H. ducreyi*; #4910 (meningococcus serogroup C) as N. *gonorrhoeae* and #4911 (Hib) as *H. ducreyi.*	-	-	-
Lab40	4/16	25%	The lab incorrectly stated #4902 (pneumococcus) was Gram-positive bacilli/coccobacilli; #4903 (*L. monocytogenes*) was Gram-positive cocci/diplococci; #4905 (meningococcus) was Gram-negative bacilli/coccobacilli. The lab identified #4906 (NTHi) as *N. meningitidis*; #4908 (meningococcus serogroup A) as "negative result"; #4909 (E. coli) as *Kingella denitrificans* and #4910 (serogroup C meningococcus) as "no growth".	-	-	-
Lab41	9/16	56%	This lab incorrectly stated #4904 (*H. influenzae*) was Gram-negative cocci/diplococci and #4905 (meningococcus) was Gram-positive cocci/diplococci. They misreported "*S. pneumoniae"* for sample #4908 (*N. meningitidis* serogroup A), and *H. influenzae* for sample #4910 (*E. coli*).	-	-	-
Lab42	12/16	75%	#4903 (*L. monocytogenes*) was incorrectly identified as Gram-negative cocci/diplococci; #4904 and #4905 were "not examined". #4906 (NTHi) was incorrectly identified as *N. meningitidis.*	-	-	-
TOTAL				2	13	14*

*As in previous years, labs are generally very good at identifying the Hib strain, but not good at positively identifying the non-Hib strain (most likely because they only stock the anti-Hib antiserum). Some correctly identified the NTHi strain, others just reported it as "not type b", but many just didn't give a result.

5.2 SSL/NL and RRL results with Full Panel:

Lab ID	Region	Gram stains	ID and typing of cultures	Detection and typing of simulated CSFs	Overall Score[1] %	Comments
RRL01	**AFRO**	**2/2 (100%)**	**26/26 (100%)**	**26/26 (100%)**	**54/54 (100%)**	Reported wrong result for *L. monocytogenes* and Hi Gram stains (not scored).
RRL02	**AFRO**	**2/2 (100%)**	**26/26 (100%)**	**24/26 (92%)**	**52/54 (96%)**	Reported wrong result for *L. monocytogenes* and Hi Gram stains (not scored). Reported typing result for the Hif positive CSF sample as non-typeable by mistake.
Lab15	**AFRO**	**2/2 (100%)**	**22/26 (85%)**	**11/26 (42%)**	**35/54 (65%)**	Reported wrong result for *L. monocytogenes* Gram stain (not scored). Did not report serogrouping results for the Nm cultures. Reported a false negative result for one of the Nm-positive CSF samples. Did not report serotyping results for the Sp-positive CSF samples. Reported the Hif positive CSF sample as non-typeable by mistake.

Lab38	**AFRO**	**0/2 (0%)**	**25/26 (96%)**	**22/26 (85%)**	**47/54 (87%)**	Reported wrong result Sp and Hi Gram stains (Hi not scored). Didn't report a result for the Nm Gram stain. Reported a partially correct serotype result for one of the Sp cultures. Was unable to type one of the Sp-positive CSF samples and one of the Nm-positive CSF samples.

6. 2019

6.1 SSLs/NLs testing the Partial Panel

Three of the four Gram stains were excluded from the scoring this year (*Hi*, Group B strep and *L. monocytogenes*).

3 labs were sent a Partial Panel, but they did not submit any results (no reason given).

2 labs were sent a Partial Panel, but they could not test the samples because they were held at customs.

LID	Score	Percentage	FULL PANEL SCORE	COMMENTS on RESULTS	Attempted optional serotyping/serogrouping of cultures?		
					Sp	Sp	Sp
Lab02	12/15	80%	79%	Reported incorrect result for all 4 Gram stains (but only 1 was scored). Didn't identify the GBS culture (stated it was a streptococcus species.)<br>Full panel<br>Failed to serotype *S.pneumoniae* #5557 (ST 3) and # 5560 (ST 19A) Failed to type # 5558 *H. influenzae* serotype a (stated it was non-b).	-	Y	Y
Lab11	10/15	67%	-	Misidentified # 5559 *(H.influenzae*) identified as *N.meningitidis*)<br>failed to identify # 5560<br>Antimicrobial susceptibility testing (not scored)<br>No errors in the 2 susceptibility results reported	-	-	-
Lab18	15/15	100%	-	No identification problems<br>Antimicrobial Susceptibility Testing (not scored)<br># 5558 (β-lactamase -ve *H.influenzae*) reported as resistant to amoxicillin-clavulanic acid<br># 5560 (S.*pneumoniae* resistant to penicillin)reported as penicillin-susceptible but oxacillin resistant. The oxacillin test indicates susceptibility/resistance to penicillin and is more reliable than using a penicillin disc.	-	Y	Y
Lab24	15/15	100%	-	No identification problems<br>Antimicrobial susceptibility testing (not scored)<br>No problems with susceptibility testing	-	Y	Y
Lab25	15/15	100%	-	No identification problems<br>Antimicrobial susceptibility testing (not scored)<br>No problems with susceptibility testing	-	-	-
Lab31	12/15	80%	-	Misidentified # 5561 (*N.meningitidis* as *Moraxella catarrhalis)*<br>Antimicrobial susceptibility testing (not scored)<br>No problems with susceptibility testing	-	Y	Y
Lab33	10/15	67%	-	Misidentified #5556 (GBS as *Enterococcus sp*) failed to identify # 5558 as *H.influenzae* (stated it was *Haemophilus sp*)<br>Antimicrobial Susceptibility testing (not scored)	-	Y	Y

				# 5560 (*S.pneumoniae* resistant to erythromycin and resistant to penicillin) reported as susceptible to penicillin and to erythromycin			
Lab34	0/15	0%	-	Misidentified # 5555 *N.meningitidis* as *S.pneumoniae, #5556 GBS* as *N.meningitidis.*<br>*# 5558 as N.meningitidis (* instead of *H.influenzae) #5560 (N.meningitidis* instead of *S.pneumoniae),*<br>*# 5561 (S.pneumoniae* instead of *N.meningitidis)*<br>Antimicrobial susceptibility testing (not scored)<br>No errors with limited testing performed	-	-	-
Lab38		93%	62%	This lab tested the Full Panel. However, they would have passed the partial panel.<br>Correctly identified all of the viable cultures. Misread Gram stain #5554 *S.pneumoniae* (GNB/CB instead of GPC/DC).<br>Full panel Failed to type *N.meningitidis*<br># 5555 (N*.meningitidis* Serogroup W) and # 5561 (*N.meningitidis* non groupable)<br>Simulated CSF samples : failed to identify both samples containing *N.meningitidis* (# 5563 and # 5568. No typing for the samples containing *H.influenzae* (# 5562 and #5564), no typing for two samples containing *S.pneumoniae* (# 5556 and #5567)	Y	Y	Y
Lab42	13/15	87%	-	No result was submitted for sample # 5558. Correct typing result for sample #5559(Hib).<br>Antimicrobial susceptibility testing (not scored)<br># 5557 (fully susceptible *S. pneumoniae*) reported oxacillin resistant.<br># 5558 β-lactamase negative ampicillin susceptible *H. influenzae*) reported as cefotaxime resistant<br>#5560 (penicillin resistant *S. pneumoniae*) reported as oxacillin sensitive	-	Y	Y
TOTAL					1	7	7

*As in previous years, labs are generally very good at identifying the Hib strain, but not good at positively identifying the non-Hib strain (most likely because they only stock the anti-Hib antiserum). Some correctly identified the Hia strain, others just reported it as “not type b”, and some just didn’t give a result.

6.2 SSL/NL and RRL results with Full Panel:

A Full panel was sent to 1 lab, but they didn’t submit any results (no reason given).

Lab No.	Region	Gram stains	ID and typing of cultures	Detection and typing of simulated CSFs	Overall Score %	Comments
RRL01	AFRO	1/1	26/26	26	**53/53 (100%)**	Reported incorrect result for Hi and *L. monocytogenes* Gram stains (not scored). No problems with cultures or CSF samples.
RRL02	AFRO	1/1	26/26	26/26	**53/53 (100%)**	Reported incorrect result for Hi and *L. monocytogenes* Gram stains (not scored). No problems with cultures or CSF samples.

Lab02	AFRO	0/1	19/26	23/26	**42 (79%)**	Reported incorrect result for Hi, GBS, *L. monocytogenes* and Sp Gram stains (only Sp scored). Could only identify the GBS culture as streptococcal species. Did not report serotyping results for Sp cultures. Could only report the Hia culture as “not type b”. Did not report a serotyping result for the 33F Sp positive CSF sample and could only type the Hia CSF sample as “non-type b”.
Lab38	AFRO	0/1	23/26	10/26	**33/53 (62%)**	Reported incorrect result for *L. monocytogenes* and Sp Gram stains (L. monocytogenes not scored). Only partially serogrouped the Nm W culture and did not correctly group the non-groupable Nm culture. Did not report any serotyping result for any of the CSF samples and did not even report a species result for the two Nm-positive CSFs.
